# Post-GDPR survey of data protection officers in research and non-research institutions in Croatia: a cross-sectional study

**DOI:** 10.11613/BM.2021.030703

**Published:** 2021-10-15

**Authors:** Anamarija Mladinić, Livia Puljak, Zvonimir Koporc

**Affiliations:** 1 Croatian Personal Data Protection Agency (AZOP), Zagreb, Croatia; 2 Center for Evidence-Based Medicine and Health Care, Catholic University of Croatia, Zagreb, Croatia

**Keywords:** ethics, research, data science, surveys, questionnaire

## Abstract

**Introduction:**

General Data Protection Regulation (GDPR) focuses on important elements of data ethics, including protecting people’s privacy, accountability and transparency. According to the GDPR, certain public institutions are obliged to appoint a Data Protection Officer (DPO). However, there is little publicly available data from national EU surveys on DPOs. This study aimed to examine the scope of work, type of work, and education of DPOs in institutions in Croatia.

**Materials and methods:**

During 2020-2021, this cross-sectional study surveyed DPOs appointed in Croatia. The survey had 35 items. The questions referred to their appointment, work methods, number and type of cases handled by DPOs, the sources of information they use, their experience and education, level of work independence, contacts with ethics committees, problems experienced, knowledge, suggestions for improvement of their work, changes caused by the GDPR, and sociodemographic information.

**Results:**

Out of 5671 invited DPOs, 732 (13%) participated in the study. The majority (91%) indicated that they could perform their job independently; they did not have prior experience in data protection before being appointed as DPOs (54%) and that they need additional education in data protection (82%).

**Conclusions:**

Most DPOs indicated that they had none or minimal prior experience in data protection when they were appointed as DPO, that they would benefit from further education on data protection, and exhibited insufficient knowledge on basic concepts of personal data protection. Requirements for DPO appointments should be clarified; mandatory education and certification of DPOs could be introduced and DPOs encouraged to engage in continuous education.

## Introduction

General Data Protection Regulation (GDPR) focuses on important elements of data ethics, including protecting people’s privacy, accountability and transparency. According to the GDPR, certain public institutions are obliged to appoint a Data Protection Officer (DPO). This applies to all public authorities, public bodies, and organizations whose main activity is the systematic and extensive monitoring of individuals or which process specific categories of personal data to a large extent, regardless of which data they process (*1*).

The DPO should take care of the protection of personal data. According to the GDPR, “The data protection officer shall be designated based on professional qualities and, in particular, expert knowledge of data protection law and practices and the ability to fulfil the tasks referred to in Article 39“ (*2*).

The GDPR has been in full force since 25 May 2018, but so far, there is little publicly available data from national EU surveys on DPOs in the post-GDPR period. For example, a survey conducted by Lopes and Oliveira between October and December 2017 found that of the four health clinics contacted, only one had appointed a DPO at the time (*3*). A study conducted on data submitted to the Croatian Personal Data Protection Agency (AZOP) showed that AZOP received only 37 opinion requests about personal data protection in research from academic and research institutions in Croatia from 2015 to 2019. For comparison, in 2018 alone, AZOP received 3464 opinion requests (*4*). A possible reason for such a small number of opinion requests related to research and personal data protection is the lack of knowledge and awareness about these issues. Another possible explanation is that researchers solve all such issues with their DPOs in their institutions. We could not find other research on this topic in the literature. The aim of this study was to examine the scope of work, type of work, and education of DPOs in institutions in Croatia.

## Materials and methods

The study was cross-sectional, conducted *via* an online survey. The protocol of the study was approved by the director of AZOP and the Ethics Committee of the Catholic University of Croatia. Detailed information about the study and personal data protection were sent to the prospective participants.

### Subjects

Eligible participants were all DPOs appointed in Croatia. At the time when the study began, the number of eligible participants (appointed DPOs) was 5671.

### Methods

Croatian Personal Data Protection Agency contacted all DPOs in Croatian institutions by e-mail and invited them to participate in the anonymous study. AZOP contacted all data protection officers whose e-mail addresses they had on file. Due to the nature of their work, AZOP needs to have contacts of all DPOs appointed in the Republic of Croatia. According to the Personal Data Protection Act of Croatia, “The Personal Data Protection Agency shall keep a Register of Personal Data Protection Officials. “ Since the AZOP has the contacts, the AZOP contacted the targeted participants.

The survey was conducted through the EU Survey platform, which did not collect respondents’ IP addresses, so participation in the survey was anonymous. After the initial invitation, the participants received three more reminders to complete the survey. In addition, DPOs attending the official AZOP workshops on data protection during November 2020 and March 2021 were also reminded to fill the survey.

For the study, we created a new survey for DPOs (Supplementary material). The new survey was created because we were unable to find such a survey in the literature. Researchers participating in the creation of the survey included experts in data protection and a research methodologist.

The survey has 35 items. The questions referred to their appointment, work methods, number and type of cases handled by DPOs, the sources of information they use, their experience and education, level of work independence, contacts with ethics committees, problems experienced, knowledge, suggestions for improvement of their work, changes caused by the GDPR, and sociodemographic information.

There were two knowledge questions. The first knowledge question asked participants to describe what are pseudonymization and anonymization. Since the participants had to explain two terms, we considered the answer partially correct if participants correctly explained only one of the two terms. The second knowledge question included the list of 10 items that must be included in the privacy policy; thus, the correct answer to this question was to choose each of the 10 items. The participants were asked to choose which of the items must be included in the privacy policy. We reported the number of participants that answered the whole question correctly (chose all 10 items as part of the privacy policy), and we also reported how many participants chose each of the 10 items.

Before sending the surveys to the respondents, a pilot test of the survey was conducted on ten trial respondents, employees of AZOP, to obtain feedback on the survey comprehensibility. The results of the pilot test were incorporated into the final version of the survey.

The survey was administered in the Croatian language. For the purpose of this manuscript, the survey was translated into English (Supplementary material). The survey was conducted between November 2020 and March 2021.

### Statistical analysis

We reported data as frequencies and percentages. We used the Shapiro-Wilk test to assess whether the distribution of continuous data was normal. Continuous data that were not normally distributed were shown as median and range.

## Results

Out of 5671 invited DPOs, 732 (13%) participated in the study. The median age of the DPOs was 42 years (704 responses); total years of lifetime employment were 15 (706 responses); the number of months serving as a DPO was 18 (703 responses). The majority were women and had a Master’s degree. Most of the DPOs in our sample were affiliated with educational institutions and public bodies. The majority (92%) were already employed by their institutions when they were appointed as a DPO (Table 1). Most DPOs (59%) did not receive a single request for an opinion from citizens/respondents who wanted to exercise their rights under the GDPR since serving as a DPO. Likewise, 83% did not receive a single complaint regarding personal data processing (Table 2).

**Table 1:** Participants’ characteristics*

**Characteristic**	**N (%)**
Age, years	42 (23-65)*
Sex	
Man	171 (23)
Woman	542 (74)
No answer	19 (2.7)
Level of education	
High school	65 (8.9)
Bachelor’s degree	117 (16)
Master’s degree	414 (57)
Specialist study	104 (14)
Scientific postgraduate study – master of science	21 (2.9)
Scientific postgraduate study – PhD	5 (0.7)
No answer	6 (0.8)
Total lifetime employment, years*	15 (0-43)
Number of months serving as a data protection officer*	18 (1-156)
Institutional affiliation	
Research institution	16 (2.2)
Educational institution	256 (35)
Government body	99 (14)
Public body	252 (34)
Private sector	88 (12)
No answer	21 (2.9)
How were you selected for the position of Data Protection Officer	
A call for recruitment of a new employee	13 (1.8)
Appointment of an existing employee to the position of DPO	674 (92)
External contractor	29 (3.9)
No answer	16 (2.2)
*Data presented as median (range).	

**Table 2:** Workload and work environment of data protection officers

**Questions/multiple-choice answers**	**N (%)**
**Number of requests received**	
None 1 to 9 10 to 50 More than 50 I cannot estimate; we do not officially file that information Another answer	431 (59) 230 (31) 49 (6.7) 0 (0) 9 (12) 13 (1.8)
**Number of complaints received**	
None 1 to 9 10 to 50 More than 50 I cannot estimate; we do not officially file that information Another answer	609 (83) 89 (12) 13 (1.8) 0 (0) 6 (0.8) 15 (2.1)
**Are you able to perform your job as a data protection officer in an independent manner?**	
Yes No No answer	668 (91) 56 (7.7) 8 (1.1)
**Do you think that you need additional education in the field of data protection?**	
Yes No No answer	597 (82) 121 (17) 14 (1.9)

There were 42 (5.7%) DPOs that indicated they received research-related questions. When asked who sent them questions related to data protection in research (multiple answers were allowed), they indicated that those individuals were administrative personnel (N = 12, 29%), junior researchers (N = 10, 24%), senior researchers (N = 6, 14%), research ethics committee (N = 5, 12%), students (N = 1, 2.4%). The majority indicated that they are able to perform their job in an independent manner (Table 2).

When asked about obstacles they encountered, 56 (7.7%) participants responded. The most common answers included: lack of independence or collaboration with their superiors (N = 28; 50%), lack of education (N = 8; 14%), and lack of time/too many obligations (N = 10; 18%).

### Education and information needs

When asked about prior experience in the field of data protection before being appointed as DPOs, 597 (82%) participants provided an answer. Among them, the majority (N = 324; 54%) did not have any prior experience; 147 (25%) indicated they had some, basic, general, or minimal experience, while 12 (1.6%) indicated they had advanced knowledge. There were 111 (19%) responses that were not possible to interpret; those participants provided responses such as “the same as now”, “positive”, “I am lawyer”, “medium”, “I worked with clients”, “I was a principal”.

Information about the formal and non-formal education that the respondents used to train/educate themselves for the position of a DPO were provided by 662 (90%) participants. The participants indicated they used: formal education (N = 210, 32%), informal education (176, 27%), education organized by AZOP (N = 87; 13%), seminars (N = 88; 13%), educations (N = 80; 12%), internet (N = 43; 6.5%), courses (N = 14; 2.1%). There were 42 (6.3%) participants that said they did not have any prior education at all. Most of the DPOs indicated that they need additional education in the field of data protection (Table 2). When participants have some issues or concerns about data protection, they indicated that they most frequently seek help or response from colleagues who are not DPOs, institutions’ legal department, and AZOP (Table 3).

**Table 3:** Frequency of seeking help or response from various information sources

**Information source**	**N (%)**					Responses, N*
	**1**	**2**	**3**	**4**	**5**
AZOP – Croatian Personal Data Protection Agency	190 (31)	56 (9.1)	98 (16)	68 (11)	202 (33)	614
Professional literature	93 (16)	75 (13)	113 (19)	128 (22)	178 (30)	587
Colleagues who are not data protection officers	245 (56)	65 (15)	67 (15)	32 (7.3)	28 (6.4)	437
Other data protection officers	186 (35)	74 (14)	100 (19)	81 (15)	84 (16)	525
Internet	68 (11)	62 (9.8)	115 (18)	128 (20)	261 (41)	634
Institution’s legal department	166 (34)	48 (16)	66 (13)	67 (14)	143 (29)	490
Sources are ranked from 1 to 5, where 1 indicates the highest frequency and 5 the lowest frequency. *Total number of participants in the study was 732; the number of respondents for each item is shown in the table						

### Knowledge questions

There were 613 participants that answered the question about the key difference between pseudonymization and anonymization; 60% answered correctly, and 13% partially correct (Table 4).

**Table 4:** Answers to knowledge questions about personal data protection

**Question/answer**	**N (%)**
**What is the key difference between pseudonymization and anonymization (N = 613)**	
Correct answer Partially correct answer Incorrect answer	365 (60) 79 (13) 169 (28)
**The privacy policy must include (N = 692)**	
Identity and contact details of the data controller/ the controller’s representative Contact of the data protection officer Legal basis for the processing The purposes for which personal data are collected and processed Recipients or categories of recipients of personal data The storage period or, if this is not possible, the criteria by which that period was determined Data subject rights	498 (72) 579 (84) 600 (87) 641 (93) 423 (61) 495 (72) 592 (86)
Information on whether the provision of personal data is a statutory or contractual requirement, or a requirement necessary to enter into a contract, as well as whether the data subject is obliged to provide the personal data and of the possible consequences of failure to provide such data	410 (60)
The existence of automated decision-making/development and meaningful information about the logic in question, as well as the importance and anticipated consequences of such processing for the respondent	242 (35)
Information on whether the data is transferred to third countries and the existence or non-existence of a European Commission adequacy decision, and if applicable, information on appropriate safeguards	411 (60)
Participants that chose all 10 items (as all must be included in the privacy policy)	162 (23)

When we asked participants to choose which of the 10 items must be included in the privacy policy, 162 (23%) of 692 who responded to that question chose all the 10 items. The most frequently chosen item was “*The purposes for which personal data are collected and processed*”, and the least frequently chosen item was “*The existence of automated decision-making/development and meaningful information about the logic in question, as well as the importance and anticipated consequences of such processing for the respondent*” (Table 4).

### Compliance with legal regulations

Most of the DPOs did not analyse personal data processing activities in their organization to comply with the data protection legislation. When they did conduct such an analysis, most of them partially made a recommendation(s) to modify the business processes to comply with the provisions of the GDPR. The majority of DPOs keep records of processing activities or participate in keeping records of processing activities. The majority never conducted a data protection impact assessment or participated in a data protection impact assessment; neither conducted nor participated in conducting a proportionality (balance) test (Table 5).

**Table 5:** Data protection officers’ compliance with legal regulations regarding personal data protection

**Regulation**	**N (%)**
**Have you conducted an analysis of personal data processing activities in your organization in order to comply with the data protection legislation?**	
Yes No Partially No answer	264 (36) 300 (41) 157 (21) 11 (1.5)
**If you have conducted an analysis of processing activities, have you made a recommendation (s) to modify the business processes in order to comply with the provisions of the GDPR?**	
Yes No Partially No answer	205 (28) 181 (25) 261 (36) 85 (12)
**Do you keep records of processing activities, or do you participate in keeping records of processing activities?**	
Yes No Not applicable No answer	379 (52) 192 (26) 144 (20) 17 (2.3)
**Have you ever conducted a data protection impact assessment or participated in a data protection impact assessment?**	
Yes No No answer	123 (17) 595 (81) 14 (1.9)
**Have you ever conducted or participated in conducting a proportionality (balance) test?**	
Yes No No answer	120 (16) 604 (83) 8 (1.1)
**In order for the controller to demonstrate reliability or compliance with the General Data Protection Regulation, it is necessary, among other things, to adopt internal data protection policies. In line with your advisory task, have you proposed the drafting and adoption of such documents?**	
Yes No No answer	441 (60) 272 (37) 19 (2.6)
**Do you think that the employees in your organization are aware of the importance of personal data protection of individuals (clients/users/employees)?**	
Yes No To a lesser degree I do not know No answer	419 (57) 56 (7.7) 213 (29) 31 (4.2) 13 (1.8)
**In your opinion, what is the biggest challenge for your organization in complying with the General Data Protection Regulation?**	
Technical data protection measures Organizational measures for the protection of personal data Insufficient level of awareness of data protection among employees Insufficient level of awareness on data protection at the management level Reporting on personal data breaches Development of regulations and data protection policies Mapping and analysis of processing procedures Data protection impact assessment Another answer	317 (43) 337 (46) 269 (37) 144 (20) 115 (16) 172 (24) 222 (30) 286 (39) 37 (5.1)
**Do you think that the GDPR has caused significant changes for data controllers/ processors and data subjects?**	
Yes, for data controllers/processors Yes, for data subjects Partly for data controllers/processors Partly for data subjects The changes are insignificant No answer	219 (30) 49 (6.7) 239 (33) 38 (5.2) 164 (22) 23 (3.1)

The majority proposed drafting and adoption of documents to adopt internal data protection policies. The respondents mostly indicated that they think that the employees in their organization are aware of the importance of personal data protection of individuals. The majority indicated that the biggest challenge for their organization in complying with the GDPR includes organizational measures for protecting personal data and technical data protection measures (Table 5). Among open-ended answers (N = 37), lack of personnel was the most common issue (N = 12; 32%). Most of the respondents indicated that they think that the GDPR has caused significant changes for data controllers/ processors (Table 5).

When asked to describe the changes induced by the GDPR, 267 (36%) responded. The most common answers were: additional work, tasks and administration (N = 142; 53%), changes regarding the rights of data subjects (N = 43; 16%), and changes regarding the data controllers (N = 26; 9.7%).

### DPOs and ethics committees

Among our respondents, 36% indicated that their institution has an ethics committee. Among those DPOs, the majority was not involved in that committee’s work in any way (Table 6). Among those that were involved, 48/263 (18%) described the following types of involvement: a member of the committee (N = 17, 35%) and counselor/advisor (N = 16, 33%), while the rest provided vague responses, for example, “cooperation when needed”.

**Table 6:** Data protection officers and ethics committees

**Question/answer**	**N (%)**
**Does your institution have an Ethics Committee or a similar body (related to ethics)?**	
Yes No I do not know No answer	263 (36) 392 (54) 68 (9.3) 9 (1.2)
**Are you involved in the work of that committee in any way?**	
Yes No	45 (6.2) 241 (33)
**Does the Ethics Committee contact you in cases when they need advice or assistance in making decisions related to personal data protection?**	
Yes, often Yes, sometimes No, never	24 (3.3) 68 (9.3) 182 (25)
**Do you consider that the Ethics Committee of your institution is competent enough to decide on issues related to the protection of personal data?**	
Yes No I do not know	86 (12) 55 (7.5) 134 (18)
**Do you consider yourself competent enough in relation to your training in the field of personal data protection and experience to be able to answer all the questions they send you to the Ethics Committee?**	
Yes No I do not know	100 (14) 55 (7.5) 127(17.4)

Most of the DPOs were never contacted by ethics committees regarding personal data protection. The majority of DPOs had less than five such contacts since their appointment. The majority indicated that they did not know whether the Ethics Committee of their institution was competent enough to decide on issues related to personal data protection. The majority of DPOs did not know whether they were competent enough to be able to answer questions sent by an Ethics Committee (Table 6). Some of those who did not find themselves competent (N = 80, 11%) indicated that the following would help them and their institution be more effective in the tasks set before them, especially by the Ethics Committee: education (N = 38, 48%), decreasing their current workload (N = 10; 13%), and experience (N = 3, 3.7%).

## Discussion

Based on our study, most DPOs in Croatia were independent in their work but indicated that their workload has increased after the introduction of GDPR. Most of the DPOs had minimal experience and knowledge of data protection, and they clearly articulated their need for further education in the field of data protection. The majority of DPOs did not fully understand their responsibilities, and they exhibited insufficient knowledge about basic concepts of data protection. DPOs had minimal interaction with ethics committees.

The introduction of GDPR appeared to be challenging for unprepared local municipalities (*1*, *5*). The unique and sensitive position of DPO under the GDPR was clearly recognized (*6*-*8*). Even though there are some EU cross-country studies aiming to produce data protection officer’s guidance, to our best knowledge, there is a paucity of publications on the role of DPOs after the period of GDPR introduction, and we were unable to find any publications involving surveys of DPOs, which would enable comparison of our results with results coming from other EU countries (*9*, *10*).

Our results indicate that the appointment of the majority of DPOs was associated with the enforcement of GDPR. Namely, the median number of months serving as DPO was 18. It is possible that many institutions have probably appointed a DPO only because they were legally obliged to do so. However, it is very concerning that the majority of surveyed DPOs claimed that they had none or minimal previous knowledge in personal data protection. According to the GDPR, Article 37 [quote]: “*The data protection officer shall be designated on the basis of professional qualities and, in particular, expert knowledge of data protection law and practices and the ability to fulfil the tasks referred to in Article 39.”* (*11*). Our survey clearly indicates that the participants did not report they had “expert knowledge” on data protection law and practices. The requirement to have “expert knowledge” is very vague, as there is no definition of the specific requirements proving such expert knowledge, such as specific prior education or years of experience in data protection. The ambiguity of this term allows institutions to appoint anyone to serve as a DPO simply to fulfil a legal obligation. This is also likely reflected in our finding that most of the DPOs were already working in their organization when they were appointed. For institutions, this must have been the easiest solution. By appointing someone within the organization to fulfil this legal request, they have fulfilled the formal requirement; however, our survey indicates that they may not have appointed the person with “expert knowledge”, or invested in the education of appointed DPOs, as most DPOs indicated they do not consider that they have sufficient knowledge in this area.

Our findings could imply that in Croatia, institutions did not efficiently use the available time from the April of 2016, when GDPR was officially announced, until the May of 2018, when GDPR came into force, to educate DPOs adequately (*1*).

Since they were appointed, most DPOs did not receive a single request for an opinion from citizens/respondents who wanted to exercise their rights under the GDPR or a single complaint regarding personal data processing. Few DPOs received research-related questions; however, participants who indicated that they work in research or educational institutions were a minority in our sample. Further studies are needed on DPOs appointed in institutions conducting research. However, even though our data were limited in this respect, they are in line with previously published data, indicating that significant aspects for data processing for scientific research purposes are not sufficiently recognized among research and academic institutions (*4*, *12*, *13*).

The majority of DPOs claimed that they were able to perform their DPO role independently. Furthermore, our survey indicated that most of the DPOs neither analysed personal data processing activities in their organization nor conducted or participated in a data protection impact assessment. To us, this indicates that most DPOs might not be fully aware of their responsibilities.

Our results also show DPO’s strong perception of workload increase, which came together with the introduction of GDPR. However, our survey did not go sufficiently into detail regarding this additional work. Since most participants indicated they did not conduct basic processes they were supposed to do, and the majority did not show basic knowledge about personal data protection, the additional workload may be associated with various aspects of their appointment.

While the participants recognized the need for additional education, this was also shown by their responses to our knowledge questions. Even though most DPOs correctly described differences between anonymization and pseudonymisation, there were still around forty percent of wrong and partially correct answers. When asked to select privacy policy items, just around twenty percent correctly chose all 10 items. That correlates with previously published assertions that DPOs positions are challenged with their insufficient knowledge of applying the GDPR (*14*).

Around one-third of DPOs responded that they have an ethics committee in their organization. Due to the anonymous nature of our study, we were unable to verify whether they indeed have or do not have such a committee in their institution. However, only a few among those DPOs received any request from their ethics committee, and the majority did not know whether they were sufficiently competent to answer potential questions of an ethics committee. A few DPOs were involved in institutional ethics committees, mostly as committee members or as administrative support. Since research usually involves many personal data protection issues, we can only hypothesize that researchers do not sufficiently recognize the importance of data protection, and this is why they seldom contact DPO or include a DPO in an ethics committee.

Our results clearly point to the need for continuous education of DPOs and a better definition of “expert knowledge” needed for a DPO appointment. We consider that our study indicates the need for some kind of standardization in DPO education. Some attempts in other EU countries to formally educate employees of public institutions were already reported (*15*).

In Croatia, as well as in the EU, many organizations offer courses for a “certified DPO”. Some of those organizations suggest that DPOs are obliged to get certification in relation to Article 42 of the GDPR. Such claims are false because the GDPR does not prescribe the certification of the DPOs nor individuals (*16*). The certification concept referred to in the Article 42 of the GDPR applies to services, products, and possibly to management systems, but not to individuals (*17*).

Some national data protection agencies (DPAs) have developed the certification schemes for DPOs. For example, French Data Protection Act provides French data protection authority (CNIL) the task of the certification for DPOs. The CNIL issued certification criteria including the list of 17 DPO skills and knowledge needed for certification, and accreditation criteria for certification bodies that would like to be accredited by the CNIL to certify skills and knowledge of DPOs in line with the criteria adopted by the CNIL (*18*). Another DPA which has developed certification scheme of DPOs is the Spanish data protection authority (*19*).

AZOP, the Croatian DPA, conducted online workshops for more than 1300 DPOs in the first four months of 2021, and the interest for this type of education increases continuously. Many of the questions DPOs ask during the workshops require basic knowledge of personal data protection, and one of the most frequently asked questions is how to certify for a DPO or does the DPO needs to be certified.

Although mandatory certification requirement for the appointment of DPOs is not prescribed by the GDPR, one option is the introduction of a voluntary certification procedure that will include standardized education by an institution such as AZOP. Our study showed that the DPOs recognize AZOP as one of the main information sources for questions related to data protection. Our study, thus, indicates that the DPOs are aware of the relevance of AZOP. Furthermore, once certified, DPOs need to be encouraged by their employers to engage in continuing education, as personal data protection issues keep evolving with the emergence of new technologies (*20*).

Personal data protection is considered an ethical issue (case in point: personal data protection is evaluated in European competitive research calls within the ethics evaluation); thus, we wanted to conduct the study in the Croatian setting that would be meaningful and would involve a large number of participants. That could be achieved only if we would target all DPOs registered in Croatia. In this study we did not focus exclusively on DPOs from research institutions or DPOs from biomedical research institutions because the number of public research institutions in the Republic of Croatia is relatively small, including just over 100 institutions. If we had targeted only research institutions with our anonymous survey, we would likely not have more than 20-30 persons in our survey, which would significantly diminish the value of our study. Furthermore, if we have decided to even further narrow down the sample and to include only those DPOs that work in the field of biomedicine, the impact of such research results would be negligible, as there are relatively few such institutions in Croatia. Due to a small number of such institutions, our survey should be considered a first step, that can help in designing future studies that will use different study design – for example qualitative studies, to gather more information on this topic from DPOs working in specific research fields.

The limitation of this study is a non-response bias, as 13% of the invited DPOs participated in the study. Furthermore, a potential limitation of the study is reliance on participants’ self-report and honesty. We had questions on knowledge, and it is possible that some of them searched for answers online or elsewhere.

In conclusion, most DPOs indicated that they had none or minimal prior experience in data protection when they were appointed as DPO, that they would benefit from further education on data protection, and exhibited insufficient knowledge on basic concepts of personal data protection. Voluntary certification of DPOS based on the standardized education, provided by the national data protection authorities, should be considered. Continuing education of DPOs needs to be encouraged. Reasons for minimal involvement of DPOs in the work of institutional ethics committees should be further explored in future studies.
