# Corrigendum to: Post-GDPR survey of data protection officers in research and non-research institutions in Croatia: a cross-sectional study

**DOI:** 10.11613/BM.2022.021201

**Published:** 2022-06-15

**Authors:** Anamarija Mladinić, Livia Puljak, Zvonimir Koporc

**Affiliations:** 1 Croatian Personal Data Protection Agency (AZOP), Zagreb, Croatia; 2 Center for Evidence-Based Medicine and Health Care, Catholic University of Croatia, Zagreb, Croatia

This is a correction of: Mladinić A, Puljak L, Koporc Z. Post-GDPR survey of data protection officers in research and non-research institutions in Croatia: a cross-sectional study. Biochem Med (Zagreb). 2021;31:030703. 10.11613/BM.2021.030703.

Since the publication of the article, the authors have noticed that one sentence was not correct, as the legislation has changed before the manuscript was published. Thus, in order to provide accurate information to the readers, the sentence: “According to the Personal Data Protection Act of Croatia, The Personal Data Protection Agency shall keep a Register of Personal Data Protection Officials.” should be disregarded. The authors apologize for any inconvenience caused to the readers.

